# Effects of candesartan on cellular redox status and bioenergetics of cardiac tissue in normal and chronically stressed rats

**DOI:** 10.3389/fphar.2026.1876050

**Published:** 2026-09-15

**Authors:** Mahmoud M. A. Abulmeaty, Hazem K. Ghneim, Dara Aldisi, Reham M. Wahid, Wesam M. R. Ashour, John P. Giesy, Mourad A. M. Aboul-Soud

**Affiliations:** 1 Department of Community Health Sciences, College of Applied Medical Sciences, King Saud University, Riyadh, Saudi Arabia; 2 Department of Clinical Laboratory Sciences, College of Applied Medical Sciences, King Saud University, Riyadh, Saudi Arabia; 3 Department of Medical Physiology, Faculty of Medicine, Zagazig University, Zagazig, Egypt; 4 Department of Veterinary Biomedical Sciences and Toxicology Centre, Western College of Veterinary Medicine, University of Saskatchewan, Saskatoon, SK, Canada; 5 Department of Integrative Biology and Center for Integrative Toxicology, Michigan State University, East Lansing, MI, United States; 6 Department of Environmental Sciences, Baylor University, Waco, TX, United States; 7 Center of Excellence in Biotechnology Research, College of Applied Medical Sciences, King Saud University, Riyadh, Saudi Arabia

**Keywords:** angiotensin receptor blocker, candesartan, cellular redox state, mitochondrial bioenergetics, myocardial tissue, oxidative stress, unpredictable chronic mild stress

## Abstract

**Background:**

Studies of redox status and bioenergetics in cardiac tissue during unpredictable chronic mild stress (UCMS) are scarce. The present study examined the effect of candesartan, an angiotensin II type 1 receptor (AT1R) blocker primarily used to treat hypertension and congestive heart failure, on the antioxidant capacity and energy homeostasis of cardiac tissue in UCMS-subjected rats.

**Methods:**

Forty-eight male Wistar rats were randomly assigned to four groups (n = 12/group): undosed normal controls (NC), UCMS-subjected rats (UCMS), unstressed candesartan-treated rats (Can), and stressed candesartan-treated rats (UCMS + Can). Enzyme activities and mRNA transcripts of key antioxidant enzymes, tricarboxylic acid cycle enzymes, and respiratory chain complexes were measured in myocardial tissue, together with oxidative-phosphorylation (OXPHOS) markers, redox state, and the energy-homeostasis ratios GSH/GSSG, NAD^+^/NADH, and ATP/ADP.

**Results:**

The UCMS group showed marked elevations in oxidative-stress markers, accompanied by significantly reduced antioxidant enzyme activities and downregulated antioxidant gene expression. In parallel, all TCA-cycle and OXPHOS enzyme activities were significantly decreased, and their relative gene transcripts were markedly downregulated. The cellular GSH/GSSG and ATP/ADP ratios were significantly lowered, whereas the NAD^+^/NADH ratio was increased. Each of these changes was either fully reversed or significantly attenuated in unstressed and stressed rats receiving candesartan.

**Conclusion:**

Candesartan clearly mitigates cellular oxidative stress and the disruption of energy metabolism in the myocardial tissue of UCMS-subjected rats. Because blood pressure, cardiac function and systemic haemodynamics were not measured, these findings should be interpreted as biochemical evidence of a cytoprotective action of AT1R blockade in myocardial tissue, and they provide a preliminary rationale for further evaluation of candesartan in stress-related cardiovascular disorders.

## 1 Introduction

Stress is a state in which homeostasis is disrupted, prompting the activation of adaptive mechanisms and behaviors that constitute the stress response to ensure survival and restore long-term equilibrium (Kyrou and Tsigos, 2009; Chu et al., 2024). Stress triggers psychological, cardiovascular, and neuroendocrine responses, including increased heart rate and the release of stress-related hormones such as cortisol, catecholamines, and angiotensin II (Ang II) (Godoy et al., 2018). Ang II is a central component of the renin–angiotensin system (RAS) and regulates blood pressure and cardiovascular equilibrium. It is generated through an enzymatic cascade and was traditionally regarded as a systemic hormone (Wen et al., 2012); more recently, Ang II is implicated in oxidative stress induction, endothelial dysfunction, vascular smooth muscle contraction, anti-apoptotic effects, and pro-mitogenic actions, which predispose to many pathophysiological states (Kanugula, et al., 2023). Persistent activation of the Ang II/AT1R axis during chronic stress has been implicated in cardiovascular, neurological, and renal disorders through mechanisms involving sustained oxidative stress, endothelial dysfunction, mitochondrial injury, and chronic inflammation (Nakagawa et al., 2020; Ajoolabady et al., 2024). Recent studies further indicate that activation of the tissue RAS contributes to adverse cardiac remodeling by promoting redox imbalance and inflammatory signaling, whereas the counter-regulatory ACE2/Ang-(1–7)/Mas receptor axis attenuates these pathological responses (Fontes et al., 2016; Oscar et al., 2015).

Ang II exerts most of its actions by binding to the angiotensin II type 1 receptor (AT1R), a membrane receptor essential for mechanosignaling in cardiac tissue (Grogan et al., 2023). Stimulation of AT1R promotes local contact formation and myocyte contraction, suggesting that the receptor mediates direct mechanical signal transduction independent of Ang II binding (Yong et al., 2016). Candesartan (2-ethoxy-1-{[2′-(1H-tetrazol-5-yl)biphenyl-4-yl]methyl}-1H-benzimidazole-7-carboxylic acid; CAS 145040-37-5) is a selective AT1R inhibitor used clinically for coronary heart disease, congestive heart failure, and, more recently, to facilitate urinary glucose excretion and protect renal function in chronic kidney disease (Zhu et al., 2023). Candesartan is administered as the cyclohexyl 1-hydroxyethyl carbonate ester (candesartan cilexetil), a prodrug that is fully metabolized by intestinal esterases during absorption to release the active candesartan moiety (Mady et al., 2021). Candesartan was reported to have a higher renoprotective effect and a lower cardioprotective effect than lisinopril in a rat model of Cardiorenal syndrome (Eldesouky et al., 2026). In addition, candesartan has been reported to exert anti-inflammatory and neuroprotective effects in the early stage of Alzheimer’s disease (Torika et al., 2018), to ameliorate the cytokine storm of COVID-19 (Elkahloun and Saavedra, 2020), and to attenuate cisplatin-induced lung injury through anti-inflammatory and antioxidant mechanisms (Atwa et al., 2022). These observations warrant further investigation at the cellular level.

Psychological stress is widely recognized as a risk factor for cardiovascular diseases (Vaccarino and Bremner, 2024). Psychosocial stress can trigger cardiac hypertrophy, hypertension, arrhythmia, ischaemic injury, and myocardial infarction (Turdi et al., 2012). Oxidative stress, defined as an imbalance between the production of reactive oxygen species (ROS)—superoxide, hydrogen peroxide, and hydroxyl radicals—and antioxidant defenses, is thought to play a central role in the oxidative damage to lipids, proteins, and DNA induced by stress. Oxidative stress is considered a hallmark of hypertension in experimental models and human research (Maes and Twisk, 2010; Camargo et al., 2025). Major sources of ROS include NAD(P)H oxidase, xanthine oxidase, and electron leakage from the mitochondrial respiratory chain. A substantial part of Ang II signalling in the vessel wall is redox-dependent, with NADPH oxidase acting as the principal enzymatic source of the ROS that transduce these effects (Koumallos et al., 2023). NAD(P)H-oxidase-derived ROS can act as an initial trigger for further ROS generation by mitochondria, a process known as “ROS-induced ROS release” (Zorov et al., 2000).

Mitochondria are recognized as central regulators of myocardial adaptation to chronic stress because they coordinate ATP production, calcium homeostasis, redox signaling, and programmed cell death. Persistent oxidative stress disrupts mitochondrial dynamics, impairs oxidative phosphorylation, decreases ATP synthesis, alters nicotinamide coenzyme metabolism, and activates apoptotic signaling pathways, ultimately contributing to cardiac dysfunction (Zong et al., 2024). Recent advances further indicate that mitochondrial dysfunction is both a consequence and an amplifier of Ang II-mediated oxidative stress, making preservation of mitochondrial integrity an important therapeutic target in cardiovascular disease (Ajoolabady et al., 2024).

Two lines of evidence are individually well established but have not been brought together. On the one hand, chronic unpredictable mild stress is known to impair cardiac mitochondrial function and to provoke myocardial oxidative injury; on the other, candesartan is an established antioxidant and anti-inflammatory agent in several organ systems. What remains unresolved is whether AT1R blockade preserves myocardial energy metabolism during chronic stress and, if so, at which level of the bioenergetic pathway that protection is exerted. Studies in both literature have typically reported isolated indices—a single antioxidant enzyme, a lipid-peroxidation marker or a global ATP measurement—rather than the coordinated behaviour of the pathway as a whole. Although recent studies have demonstrated that AT1R blockade attenuates oxidative stress, inflammation, and adverse cardiac remodeling, relatively few investigations have comprehensively examined its effects on myocardial mitochondrial bioenergetics during chronic unpredictable mild stress. In particular, the coordinated regulation of mitochondrial nicotinamide coenzymes, tricarboxylic acid (TCA) cycle enzymes, respiratory-chain complexes, antioxidant defense systems, and their corresponding gene expression profiles following AT1R inhibition remains poorly understood. The present study was therefore designed to fill this specific gap. It profiles, in the same animals and in the same myocardial preparations, both the enzymatic activities and the corresponding transcript levels of the antioxidant defence system, the complete set of TCA-cycle enzymes, the respiratory-chain complexes, and the nicotinamide and adenine nucleotide redox couples, under AT1R blockade during UCMS. This paired enzymatic-and-transcriptional mapping allows the level at which candesartan acts within cardiac energy metabolism to be localised rather than inferred, and constitutes the principal novelty of the present work.

## 2 Materials and methods

### 2.1 Animals and experimental design

Forty-eight male Wistar rats (190–230 g) were obtained from the animal house of the Faculty of Veterinary Medicine, Zagazig University. Animals were housed under a 12 h light/12 h dark cycle (lights on 06:00, lights off 18:00) at 25 °C, with *ad libitum* access to standard rodent chow and water. After 1 week of acclimatization, rats were randomly divided into four groups (n = 12/group): (1) Normal control (NC), which received an oral gavage of 1 mL of 0.5% methylcellulose solution and was not exposed to stress; (2) Unpredictable chronic mild stress (UCMS), in which rats received an oral gavage of 1 mL of 0.5% methylcellulose solution and were exposed to alternating mild stressors for 28 days (Willner et al., 1987; Burstein et al., 2017). Stressors comprised a combination of physical and social challenges: cage tilting at 45° for 24 h, wet bedding for 4 h, continuous light exposure for 24 h, exposure to warm air for 10 min, restraint in narrow tubes for 1 h, agitation at 150 rpm for 1 h, food or water deprivation for 24 h, and social isolation for 30 min. To prevent habituation, the type and order of stressors were varied daily for 28 days (3) Candesartan-treated (Can), which comprised unstressed rats receiving daily candesartan cilexetil (Atacand®, AstraZeneca, Sweden) at 0.1 mg/kg by oral gavage (Braszko et al., 2013); and (4) UCMS plus candesartan (UCMS + Can), in which UCMS rats received the same daily oral dose of candesartan 30 min before stressor application. At the end of the intervention, blood samples were taken from the tail vein, and plasma corticosterone was measured by ELISA (MyBiosource, United States; catalog MBS761865) to confirm establishment of the UCMS model.

Plasma corticosterone was chosen as the primary index of successful UCMS induction because elevation of circulating corticosterone is the most widely used biochemical confirmation of chronic-stress load in this paradigm (Willner, 2017), and the concurrent body-mass trajectory (Figure 1A) served as a supporting physiological index. Because corticosterone rises acutely at the onset of each individual stressor, the timing of sampling relative to the last stressor is critical (Willner, 2017); all samples were therefore obtained under the single standardised schedule described in Section 2.2. We acknowledge that the behavioural readouts conventionally applied to UCMS—sucrose-preference testing, coat-state and grooming scores, open-field and forced-swim testing—were not performed. The present protocol was designed around terminal myocardial biochemistry rather than behavioural phenotyping, and repeated behavioural testing would itself have imposed an additional and uncontrolled handling load on the NC and Can groups. This is acknowledged explicitly as a limitation in Section 4.1.

**FIGURE 1:**
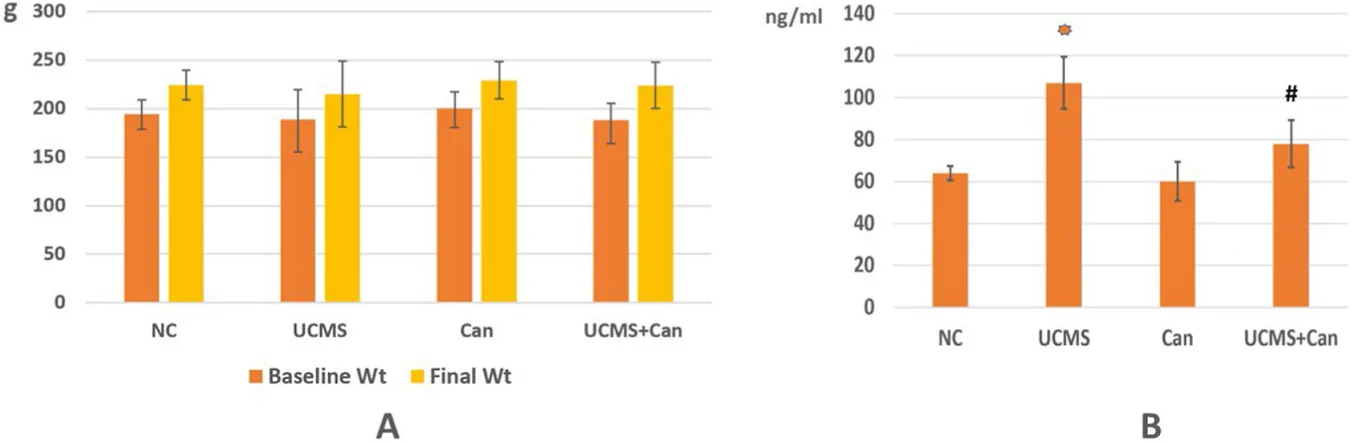
Body mass and plasma corticosterone in the four study groups. **(A)** Body mass at baseline (open bars) and at the end of the 28-day intervention (filled bars) in each group; neither baseline body mass nor the baseline-to-endpoint gain differed significantly between groups (adjusted P > 0.05 for all comparisons). **(B)** Plasma corticosterone measured at the end of the 28-day UCMS protocol. Bars represent mean ± SD (n = 12 per group). * indicates a significant difference versus the NC group (P < 0.001 for UCMS vs. NC); # indicates a significant difference versus the UCMS group (P < 0.001 for UCMS + Can vs. UCMS).Group summary statistics are reported for this panel in Supplementary Table S2.

The study protocol was reviewed and approved by the Institutional Animal Care and Use Committee, College of Sciences, Zagazig University (reference ZU-IACUC/3/F/81/2021, dated 27 June 2021).

In accordance with the approved protocol, humane endpoints and exclusion criteria were defined *a priori* in consultation with the attending institutional veterinarian. Animals were inspected daily throughout the 28-day protocol and were to be withdrawn and humanely euthanised if any of the following were observed: loss of more than 20% of baseline body mass; open, infected or self-inflicted wounds; inability to reach food or water; persistent hunched posture, piloerection with immobility, or laboured respiration; or any other condition judged by the veterinarian to cause more than transient distress. No animal met an exclusion criterion during the study and all 48 rats completed the protocol, so that 12 animals per group were available for analysis.

### 2.2 Tissue sampling

All rats were euthanized between 8 and 11 a.m., 1 day after the last stressor and last gavage. After euthanasia with a ketamine (85 mg/kg) and xylazine (3 mg/kg) mixture, the thoracic cage was opened, and the whole heart was excised, placed in cryotubes, and immediately frozen. Tissue samples were transported under strict cold-chain conditions to the laboratory of the Department of Clinical Laboratory Sciences, College of Applied Medical Sciences, King Saud University, and stored at −80 °C until analysis.

### 2.3 Isolation of cardiac mitochondria

Cardiac mitochondria were isolated by differential centrifugation as described previously, with minor modifications (Frezza et al., 2007; Ghneim and Al-Sheikh, 2010; Abulmeaty et al., 2023). Briefly, frozen myocardial tissue was thawed on ice, finely minced, and homogenized in ice-cold mitochondrial isolation buffer (250 mM sucrose, 10 mM Tris–HCl, 1 mM EGTA, and 0.5% (w/v) fatty-acid-free bovine serum albumin, pH 7.4) using a Potter–Elvehjem homogenizer fitted with a Teflon pestle (10 strokes on ice). The homogenate was centrifuged at 1,000 × g for 10 min at 4 °C to sediment nuclei, unbroken cells, and tissue debris. The supernatant was carefully decanted and centrifuged at 10,000 × g for 20 min at 4 °C to pellet the crude mitochondrial fraction. The mitochondrial pellet was washed twice by resuspension in the same buffer lacking albumin, followed by re-centrifugation at 10,000 × g for 20 min at 4 °C. The final pellet was resuspended in a small volume of ice-cold storage buffer (250 mM sucrose, 10 mM Tris–HCl, pH 7.4), and the mitochondrial protein concentration was determined by the Bradford method using bovine serum albumin as the standard. The isolated mitochondrial suspensions were used to determine ATP and ADP levels, tricarboxylic acid (TCA) cycle enzyme activities, mitochondrial nicotinamide coenzymes (NAD^+^ and NADH), and respiratory-chain complex activities. The purity and integrity of the mitochondrial preparation were verified by the latency of citrate synthase, a mitochondrial matrix marker enzyme.

### 2.4 Antioxidant enzymes and oxidative-stress markers

Activities of glutathione peroxidase 1 (GPx), superoxide dismutase (SOD), catalase (CAT), glutathione reductase (GR), glutamate–cysteine ligase (GCL), and glutathione synthetase (GS) were measured spectrophotometrically in myocardial tissue sonicates. In addition, generation rates and tissue concentrations of redox-status and oxidative-stress markers—reduced and oxidized glutathione (GSH and GSSG) and the GSH/GSSG ratio, lipid peroxides (LPO), superoxide anion (O_2_·^-^), hydrogen peroxide (H_2_O_2_), total protein carbonyl content (PCC), peroxynitrite (ONOO^−^), and nitric oxide (NO)—were determined spectrophotometrically using procedures we have described previously (Ghneim, 2017; Ghneim and Alshebly, 2016; Abulmeaty et al., 2023).

### 2.5 Mitochondrial nicotinamide coenzyme assays

Aliquots of the isolated cardiac mitochondrial suspension prepared as described in Section 2.3 were extracted on ice in 0.1 M NaOH or 0.1 M HCl to recover reduced (NADH) and oxidized (NAD^+^) nicotinamide adenine dinucleotide, respectively. Extracts were neutralized to pH 7.5 (NADH) or pH 6.5 (NAD^+^). Glycylglycine buffer (2 mL, pH-matched) was added, and samples were centrifuged at 10,000 × g for 20 min at 4 °C. Aliquots of NAD^+^ and NADH supernatants containing 30 µg of mitochondrial protein were mixed with a reaction medium containing 50 mM glycylglycine, 0.5 mM phenazine ethosulfate, 0.5 mM EDTA, 1 mM thiazolyl blue, and alcohol dehydrogenase (65 μg/mL). The reaction was initiated with 0.6 M ethanol, and the increase in absorbance was followed at 570 nm (Abulmeaty et al., 2023; Ghneim et al., 2015).

### 2.6 Mitochondrial ATP and ADP assays

Adenosine triphosphate (ATP) levels in isolated cardiac mitochondria were determined using a colorimetric assay kit (Solarbio Life Science, Beijing, China). ATP was converted to ADP by hexokinase during the phosphorylation of glucose to glucose-6-phosphate, which was subsequently dehydrogenated by glucose-6-phosphate dehydrogenase. The coupled reduction of NADP^+^ to NADPH was monitored at 340 nm and used to calculate ATP concentrations. ADP was determined by inducing its conversion to ATP and pyruvate, with absorbance read at 570 nm. ATP and ADP levels were expressed as nmol/mg mitochondrial protein, and the ATP/ADP ratio was calculated (Ghneim and Al-Sheikh, 2010).

### 2.7 Tricarboxylic-acid cycle and respiratory-chain complex assays

Aliquots of the isolated cardiac mitochondrial suspension (containing 20 µg mitochondrial protein) were used to measure the activities of TCA-cycle enzymes—citrate synthase (CS), α-ketoglutarate dehydrogenase (α-KGDH), NAD^+^-dependent isocitrate dehydrogenase (NAD^+^-ICDH), succinate dehydrogenase (SDH), and malate dehydrogenase (MDH)—using established spectrophotometric assays (Abulmeaty et al., 2023; Ghneim et al., 2015). Activities were expressed in nmol or µmol/min/mg mitochondrial protein.

Activities of mitochondrial respiratory chain complexes I–III, II–III, and IV were measured in the same isolated mitochondrial preparations. Complex I–III activity was determined by following the reduction of cytochrome c at 550 nm. Complex II–III activity was assayed by adding the mitochondrial suspension to a reaction medium containing 100 µM cytochrome c and 10 mM succinate and recording the absorbance at 550 nm. Complex IV activity was followed as the rate of cytochrome c oxidation at 550 nm (Ghneim and Al-Sheikh, 2010).

### 2.8 Gene expression analysis

Total RNA was isolated from whole myocardial tissue (not from the isolated mitochondrial fraction) using the RNeasy Mini Kit (Qiagen, Hilden, Germany), and purity was checked by Nanodrop spectrophotometry. Approximately 1 µg of RNA was heat-denatured and reverse-transcribed using the RevertAid First Strand cDNA Synthesis Kit (Thermo Fisher Scientific, Waltham, MA, United States). Specific primer sets for *rnGpx1, rnSod2, rnCat, rnGsr, rnGclc, rnGss, rnCs, rnOgdh, rnIdh3a, rnSdha, rnMdh2, rnNdufs1, rnSdhb, rnCox4i1,* and *ACTB* were obtained from BioMol Laboratories (Hamburg, Germany) and used with PowerUp™ SYBR™ Green Master Mix (Applied Biosystems, Carlsbad, CA, United States) on an iQ™5 Multicolor Real-Time PCR Detection System (Bio-Rad, Hercules, CA, United States). Relative gene expression was calculated as fold change normalized to *ACTB* (housekeeping gene) using the 2^−ΔΔCT^ method. The nucleotide sequences (5′→3′) of the forward and reverse primers used for each target gene, together with the official gene symbol, full gene name and RefSeq accession of every target, are listed in Supplementary Table S1. Because Complex I–III, Complex II–III and Complex IV denote combined or multi-subunit enzymatic activities rather than single genes, each was represented at the transcript level by a single nuclear-encoded subunit: *Ndufs1* for Complex I–III, *Sdhb* for Complex II–III and *Cox4i1* for Complex IV. *Sdha* (succinate dehydrogenase flavoprotein subunit A, reported here as SDH) and *Sdhb* (succinate dehydrogenase iron–sulfur subunit B, reported here as Complex II–III) are two distinct genes and are labelled separately throughout the text, Figure 2 and Supplementary Table S1.

**FIGURE 2:**
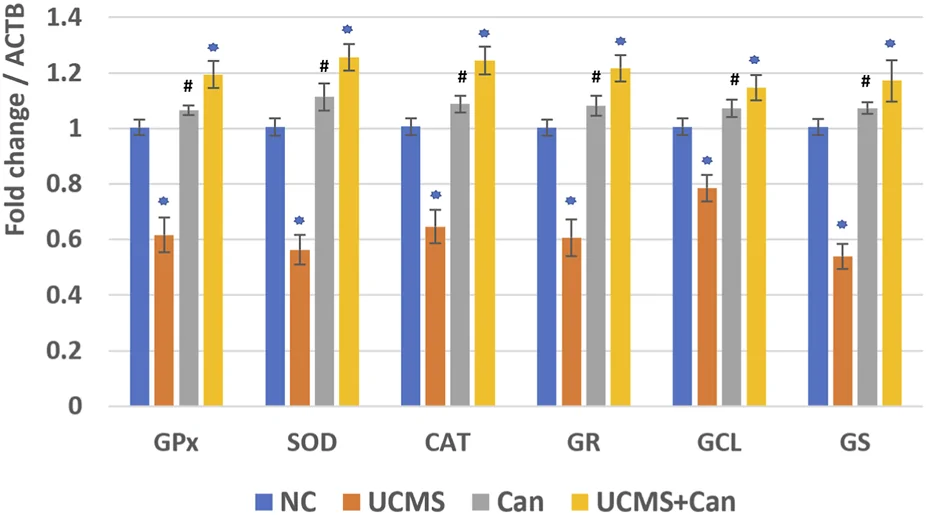
Fold change in the gene expression of TCA-cycle enzymes and respiratory-chain complex subunits in myocardial tissue of the four study groups. Transcripts shown are *Cs* (*CS*), *Ogdh (α-KGDH)*, *Idh3a (NAD* ^*+*^ *-ICDH), Sdha (SDH), Mdh2 (MDH), Ndufs1 (Complex I–III), Sdhb (Complex II–III) and Cox4i1 (Complex IV)*; *Sdha and Sdhb* are distinct genes. Primer sequences and accessions for all targets are given in Supplementary Table S1. Bars represent mean ± SD (n = 12 per group). * indicates a significant difference versus the NC group; # indicates a significant difference between the Can group and the NC group (P < 0.05). Group summary statistics (Mean ± SD) are provided in Supplementary Table S2.

### 2.9 Statistical analysis

Continuous data are presented as mean ± standard deviation (SD). Normality was assessed with the Shapiro–Wilk test. One-way analysis of variance (ANOVA) was used to compare group means. Three pairwise comparisons were pre-specified for every variable—NC vs. UCMS (effect of stress), NC vs. Can (effect of candesartan in unstressed rats) and UCMS vs. UCMS + Can (effect of candesartan under stress)—and were tested *post hoc* using the pooled within-group error term. To control the family-wise error rate, the resulting P-values were adjusted for the three planned comparisons using the Šidák correction; exact adjusted P-values are reported for each comparison in Tables 1 and 2 rather than an omnibus P-value alone. A two-tailed adjusted P-value ≤0.05 was considered statistically significant. All analyses were performed using SPSS version 23 (SPSS Inc., Chicago, IL, United States).

**TABLE 1:** Antioxidant enzyme activities, reduced/oxidized glutathione, and oxidative-stress markers in myocardial tissue of the four study groups.

Variable	NC (n = 12)	UCMS (n = 12)	Can (n = 12)	UCMS + Can (n = 12)	Adj. P NC vs. UCMS	Adj. P NC vs. Can	Adj. P UCMS vs. UCMS + Can
Antioxidant enzyme activity							
GPx (nmol/min/mg protein)	565.25 ± 56.11	353.17 ± 40.62	602.5 ± 37.5	671.42 ± 63.93	<0.001	0.219	<0.001
SOD (µmol/min/mg protein)	37.08 ± 4.93	22.04 ± 3.2	40.66 ± 4.16	46.5 ± 4.67	<0.001	0.135	<0.001
CAT (µmol/min/mg protein)	47.9 ± 5.11	30.83 ± 4.72	51.8 ± 5.22	58.91 ± 6.15	<0.001	0.221	<0.001
GR (µmol/min/mg protein)	7.04 ± 0.71	4.55 ± 0.48	7.59 ± 0.62	8.56 ± 0.84	<0.001	0.149	<0.001
GCL (µmol/min/mg protein)	14.34 ± 0.95	11.47 ± 0.74	15.12 ± 0.94	16.38 ± 1.02	<0.001	0.125	<0.001
GS (µmol/min/mg protein)	356.33 ± 28.5	188.83 ± 26.17	380.33 ± 28.31	416.42 ± 33.31	<0.001	0.143	<0.001
Reduced/oxidized glutathione							
GSH (nmol/mg tissue)	29.48 ± 3.9	18.65 ± 2.17	32.44 ± 4.04	37.76 ± 4.65	<0.001	0.193	<0.001
GSSG (nmol/mg tissue)	1.81 ± 0.22	3.02 ± 0.3	1.67 ± 0.16	1.48 ± 0.13	<0.001	0.304	<0.001
GSH/GSSG ratio	16.54 ± 2.91	6.22 ± 0.96	19.53 ± 2.47	25.76 ± 4.81	<0.001	0.067	<0.001
Reactive oxygen species and oxidative-stress markers							
Lipid peroxides (nmol/g tissue)	141.16 ± 10.71	315.48 ± 55.31	132.25 ± 10.49	119.08 ± 9.71	<0.001	0.840	<0.001
Superoxide anion (µmol/min/mg protein)	3.45 ± 0.38	6.21 ± 0.48	3.2 ± 0.23	2.93 ± 0.19	<0.001	0.219	<0.001
Hydrogen peroxide (nmol/min/mg protein)	26.87 ± 4.15	60.64 ± 13.27	24.2 ± 3.37	20.45 ± 2.84	<0.001	0.756	<0.001
Total protein carbonyl content (nmol/g tissue)	2.36 ± 0.46	5.8 ± 0.51	2.05 ± 0.38	1.65 ± 0.3	<0.001	0.215	<0.001
Peroxynitrite radical (µmol/g tissue)	644.42 ± 38.38	914 ± 40.24	618.75 ± 35.72	575.75 ± 33.6	<0.001	0.264	<0.001
Nitric oxide (µmol/g tissue)	1.21 ± 0.18	3.24 ± 0.39	1.09 ± 0.14	0.94 ± 0.11	<0.001	0.512	<0.001

GPx, glutathione peroxidase; SOD, superoxide dismutase; CAT, catalase; GR, glutathione reductase; GCL, glutamate–cysteine ligase; GS, glutathione synthetase; GSH, reduced glutathione; GSSG, oxidized glutathione. Values are mean ± SD (n = 12 per group). Exact P-values are provided. Groups were compared by one-way ANOVA, followed by the three pre-specified pairwise contrasts shown; P-values are two-tailed and Šidák-adjusted for these three comparisons. Adjusted P ≤ 0.05 was considered significant. The omnibus ANOVA P-value was <0.001 for every variable in this table.

**TABLE 2:** ATP/ADP levels, TCA-cycle enzymes, mitochondrial nicotinamide coenzymes, and respiratory-chain complexes in myocardial tissue of the four study groups.

Variable	NC (n = 12)	UCMS (n = 12)	Can (n = 12)	UCMS + Can (n = 12)	Adj. P NC vs. UCMS	Adj. P NC vs. Can	Adj. P UCMS vs. UCMS + Can
ATP/ADP levels							
ATP (nmol/mg mitochondrial protein)	170.12 ± 10.07	92.29 ± 5.23	181.02 ± 9.17	200.74 ± 10.3	<0.001	0.026	<0.001
ADP (nmol/mg mitochondrial protein)	14.68 ± 1.03	28.03 ± 2.54	13.61 ± 0.99	13.85 ± 1.29	<0.001	0.288	<0.001
ATP/ADP ratio	11.65 ± 1.07	3.32 ± 0.38	13.39 ± 1.45	14.61 ± 1.5	<0.001	0.002	<0.001
TCA-cycle enzymes							
Citrate synthase (nmol/min/mg mitochondrial protein)	1.32 ± 0.08	0.53 ± 0.08	1.41 ± 0.08	1.56 ± 0.09	<0.001	0.032	<0.001
α-Ketoglutarate dehydrogenase (nmol/min/mg mitochondrial protein)	47.71 ± 5.85	29.13 ± 2.25	53.58 ± 4.4	59.43 ± 2.58	<0.001	0.003	<0.001
NAD+-dependent isocitrate dehydrogenase (nmol/min/mg mitochondrial protein)	20.93 ± 2.38	9.77 ± 1.09	23.95 ± 2.09	28.11 ± 1.4	<0.001	<0.001	<0.001
Succinate dehydrogenase (nmol/min/mg mitochondrial protein)	70.49 ± 4.83	42.88 ± 2.67	75.36 ± 3.44	80.95 ± 1.93	<0.001	0.003	<0.001
Malate dehydrogenase (µmol/min/mg mitochondrial protein)	8.85 ± 0.92	5.54 ± 0.49	10.31 ± 1.19	12.82 ± 0.72	<0.001	<0.001	<0.001
Mitochondrial nicotinamide coenzymes							
NAD+ (pmol/mg mitochondrial protein)	455.83 ± 27.19	192.72 ± 11.3	489.93 ± 39.79	516.19 ± 16.45	<0.001	0.008	<0.001
NADH (pmol/mg mitochondrial protein)	61.09 ± 5.66	23 ± 3.06	68.32 ± 6.79	77.58 ± 4.46	<0.001	0.004	<0.001
NAD+/NADH ratio	7.54 ± 0.95	8.49 ± 0.71	7.21 ± 0.71	6.68 ± 0.51	0.009	0.624	<0.001
Mitochondrial respiratory-chain complexes							
Complex I–III (nmol/min/mg mitochondrial protein)	1.46 ± 0.08	0.72 ± 0.09	1.56 ± 0.09	1.65 ± 0.07	<0.001	0.015	<0.001
Complex II–III (nmol/min/mg mitochondrial protein)	2.08 ± 0.15	0.82 ± 0.02	2.31 ± 0.2	2.56 ± 0.14	<0.001	0.007	<0.001
Complex IV (nmol/min/mg mitochondrial protein)	702.92 ± 20.63	363.67 ± 21.8	724.25 ± 12.07	754.08 ± 13.85	<0.001	0.032	<0.001

ATP, adenosine triphosphate; ADP, adenosine diphosphate; NAD^+^, oxidized nicotinamide adenine dinucleotide; NADH, reduced nicotinamide adenine dinucleotide; Complex I–III, NADH–cytochrome c reductase; Complex II–III, succinate–cytochrome c reductase; Complex IV, cytochrome c oxidase. All parameters in this table were measured in isolated cardiac mitochondria and are normalized to mitochondrial protein. Values are mean ± SD (n = 12 per group).Exact P-values are provided. Groups were compared by one-way ANOVA, followed by the three pre-specified pairwise contrasts shown; P-values are two-tailed and Šidák-adjusted for these three comparisons. Adjusted P ≤ 0.05 was considered significant. The omnibus ANOVA P-value was <0.001 for every variable in this table.

## 3 Results

### 3.1 Body-mass changes and plasma corticosterone

Body mass at baseline and at the end of the 28-day intervention is shown for each group in Figure 1A. Neither baseline body mass nor the baseline-to-endpoint gain in body mass differed significantly between groups. Plasma corticosterone at the end of the interventions was significantly elevated in the UCMS group, confirming the establishment of chronic stress in the UCMS model (Figure 1B). Plasma corticosterone in the UCMS + Can group was significantly lower than in the UCMS group.

### 3.2 Effects of candesartan on myocardial tissue in unstressed rats

Compared with the NC group, candesartan alone produced a consistent but statistically non-significant trend towards higher antioxidant enzyme activity, the largest differences being for SOD, GCL and the GSH/GSSG ratio (adjusted P = 0.135, 0.125 and 0.067, respectively; Table 1). After adjustment for the three planned comparisons, no antioxidant enzyme, glutathione measure or oxidative-stress marker differed significantly between the Can and NC groups. ATP concentration and the ATP/ADP ratio were significantly higher in the Can group than in the NC group (adjusted P = 0.026 and 0.002; Table 2). Candesartan treatment also significantly increased all measured TCA-cycle enzymes (CS, α-KGDH, NAD^+^-ICDH, SDH, and MDH; Table 2). In addition, mitochondrial NAD^+^ and NADH and the activities of the respiratory chain complexes were significantly higher in the Can group than in the NC group. Gene-expression results (Figures 2, 3) showed that fold changes of *rnGpx1, rnSod2, rnCat, rnGsr, rnGclc, rnGss, rnCs, rnOgdh, rnIdh3a, rnSdha, rnMdh2, rnNdufs1* (Complex I–III), *rnSdhb* (Complex II–III) and *rnCox4i1* (Complex IV) were significantly higher in the Can group than in the NC group (P < 0.05 for all comparisons).

**FIGURE 3:**
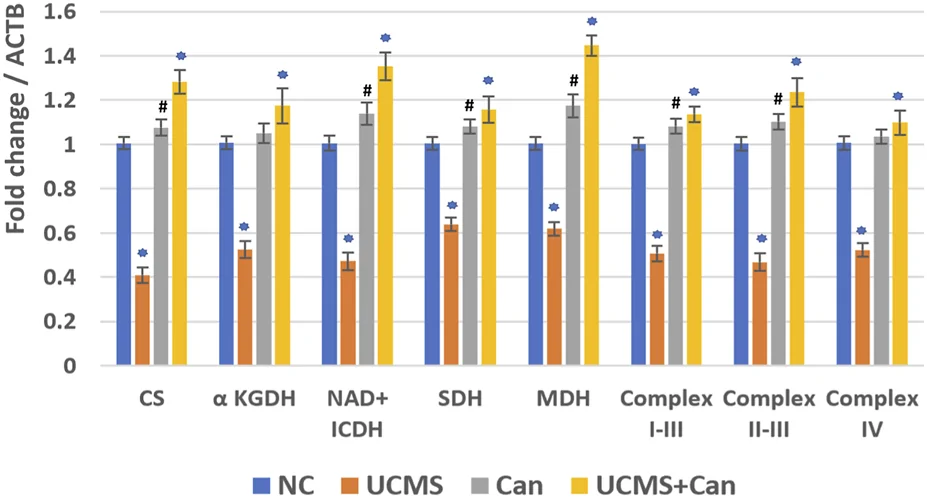
Fold change in the gene expression of antioxidant enzymes in myocardial tissue of the four study groups. Bars represent mean ± SD (n = 12 per group). * indicates a significant difference versus the NC group; # indicates a significant difference between the Can group and the NC group (P < 0.05). Individual values (Mean ± SD) are provided in Supplementary Table S2.

### 3.3 Myocardial tissue changes in rats exposed to chronic stress

Compared with the NC group, UCMS-exposed rats showed marked reductions in the activities of all antioxidant enzymes studied, lower GSH levels, higher GSSG levels, and elevated concentrations of all ROS and oxidative-stress markers (Table 1). UCMS rats also showed significantly lower ATP concentrations, higher ADP concentrations, reduced TCA-cycle enzyme activities, lower mitochondrial nicotinamide coenzyme levels, and decreased respiratory-chain complex activities (Table 2). Gene expression analyses (Figures 2, 3) confirmed the deleterious effects of UCMS on myocardial tissue.

### 3.4 Effects of candesartan on myocardial tissue in stressed rats

Compared with the UCMS group, UCMS + Can rats showed significant increases in the activities of GPx, SOD, CAT, GR, GCL, and GS. GSH was significantly higher, and GSSG was significantly lower, resulting in a significantly higher GSH/GSSG ratio (Table 1; P < 0.001 for all comparisons). All oxidative-stress markers were significantly lower in the UCMS + Can group than in the UCMS group (P < 0.01 for all comparisons), reflecting amelioration of UCMS-induced redox disturbances. Comparable or even more favorable values were obtained when the UCMS + Can group was compared with the NC group. ATP concentrations were significantly higher and ADP concentrations significantly lower in the UCMS + Can group than in the UCMS group, yielding a higher ATP/ADP ratio (Table 2). TCA-cycle enzyme activities, mitochondrial NAD^+^ and NADH, and the activities of the respiratory chain complexes were also significantly higher in the UCMS + Can group than in the UCMS group (P < 0.01 for all comparisons). Gene-expression results (Figures 2, 3) supported these beneficial changes.

## 4 Discussion

The results presented here characterize the effects of low-dose candesartan on myocardial tissue in normal rats and in rats exposed to UCMS, focusing on cellular antioxidant enzyme activities, redox status, and bioenergetics, with the aim of identifying possible cellular mechanisms of candesartan’s cardiac actions. In unstressed rats, pre-treatment with candesartan reduced markers of oxidative stress, enhanced TCA-cycle enzyme activities, and increased ATP concentration and the ATP/ADP ratio. These findings are consistent with previous evidence that candesartan exerts antifibrotic and antioxidant effects *in vitro* and *in vivo* (Zhu et al., 2023). Candesartan has also been reported to induce microvascular growth in skeletal and cardiac muscle in healthy humans, an effect attributed to a relative shift of Ang II signaling toward the angiotensin type 2 receptor (AT2R) following selective AT1R blockade (Sauder et al., 2012). The observed support of TCA-cycle activity and improvement in glucose handling may help to explain why AT1R blockade has been associated with a lower risk of incident diabetes; in the VALUE trial, long-term valsartan use was associated with a lower incidence of new-onset diabetes than amlodipine (Julius et al., 2004), and candesartan has been shown to reduce the development of diabetes in patients with heart failure (Yusuf et al., 2005).

In the present study, ATP concentration and the ATP/ADP ratio were higher in unstressed candesartan-treated rats than in normal controls. Candesartan-induced increases in ATP may be released from cardiomyocytes by exocytosis, thereby raising extracellular ATP (Vultaggio-Poma et al., 2020). Extracellular ATP can stimulate purinergic P2 receptors on the cell membrane, enhance glucose-stimulated insulin secretion, and modulate hepatic glucose and lipid metabolism (Li et al., 2022). Of note, candesartan exposure was previously reported not to alter plasma insulin, glucose, or free-fatty-acid concentrations, nor the response of free fatty acids to insulin during euglycaemic clamp (Sauder et al., 2012). Acute myocardial ischemia–reperfusion injury is another setting in which oxidative stress, inflammation, and redox status are critical determinants of outcome (Kurian et al., 2016). The amelioration of ROS and oxidative-stress markers by candesartan is consistent with its reported cardioprotective effect in ischemia–reperfusion injury (Sheik Uduman et al., 2016). AT1R blockers have also been reported to exert anti-inflammatory effects and to reduce cerebral injury after stroke (Al-Nimer, 2012).

Chronic stress in humans, modeled by UCMS in rodents, is associated with multiple cardiovascular disorders. Increased cardiac expression of serotonin (5-HT) and growth-associated protein-43 has been reported in UCMS-exposed rats and has been linked to a higher risk of cardiovascular disease (Liu et al., 2018). UCMS has been shown to cause myocardial injury, abnormal systolic and diastolic function, increased oxidative stress, and inflammation in mouse hearts (Zhang et al., 2023). Hemodynamic alterations have also been described, including increased heart rate, elevated cardiac sympathetic tone, and reduced heart rate variability, without a significant change in mean arterial pressure (Grippo et al., 2003). The current study showed that UCMS reduced GPx, SOD, CAT, GR, GCL, GS, and GSH and markedly increased ROS, with concomitant reductions in TCA-cycle enzymes and respiratory-chain complexes—a profile that may predispose to cardiovascular disease. These findings are partially consistent with a previous study in which 9.5 weeks of UCMS increased myocardial lipid peroxidation but failed to alter cardiac SOD, CAT, or total GSH compared with controls (Geddie et al., 2023); the shorter, 4-week UCMS protocol used here, together with a different stressor schedule, may explain the more pronounced enzymatic changes we observed. UCMS-induced oxidative stress and redox disturbance have also been reported in liver, pancreas, and adipose tissue, with reductions in GSH, SOD, and CAT, increases in TNF-α, IL-6, and IL-1β, and lower IL-10 (López-López et al., 2016; Abulmeaty et al., 2023).

Pre-treatment with candesartan protected against UCMS-induced deterioration in cellular redox status and reduction in cardiac tissue bioenergetics. Increased Ang II levels associated with chronic stressful conditions act via AT1R to stimulate vascular smooth muscle proliferation, cardiac hypertrophy, and fibrosis. Conversely, AT2R antagonizes these effects, resulting in anti-inflammatory, antifibrotic, and antioxidative effects. Besides, Ang II mediates vascular hyperpermeability and expression of adhesion molecules, leading to inflammation and vascular injury (Valentini et al., 2025). Nuclear factor κB signalling is a central node in this process: inappropriate activation of the pathway drives transcription of pro-inflammatory cytokines and amplifies ROS production, and sustained activity of this axis has been linked to endothelial injury, myocardial damage and progression of atherosclerotic lesions (Liu et al., 2026). Our findings support the proposition that elevated Ang II activity contributes to the cardiovascular consequences of chronic stress and add to the evidence that AT1R blockade by candesartan can attenuate UCMS-associated cardiac disturbance and may be relevant to the future prevention of the cardiovascular consequences of stress-related disorders such as anxiety and depression (Gong and Deng, 2023). Besides, candesartan cilexetil (at a dose of 3.0 mg/kg per day) showed a cardioprotective effect against pressure overload-induced adverse electrical remodeling in rats by normalizing depressed transient outward potassium efflux and preserving mRNA levels of both voltage-gated potassium 4.2 and 4.3 channel subunits, as well as improving calcium handling, leading to a reduction in the susceptibility to tachyarrhythmias (Chang et al., 2022).

### 4.1 Limitations

This study has some limitations. First, only male rats were studied; sex-specific differences in stress responses and in the response to AT1R blockade were therefore not addressed. Second, a single dose of candesartan was used; dose–response relationships and the effects of post-stress (rather than pre-treatment) administration warrant further investigation. Third, although enzyme activities and gene expression were both measured, protein-level expression of antioxidant and bioenergetic targets was not assessed by Western blot. Fourth, cardiac functional parameters, histology sections, fibrosis markers, cardiomyocyte injury markers, echocardiography, or hemodynamic parameters were not investigated. Finally, behavioral readouts of anxiety- and depression-like phenotypes were not included; future work should integrate behavioral, biochemical, and molecular endpoints in the same animals. Establishment of the UCMS model was therefore confirmed biochemically, by the elevation of plasma corticosterone, and physiologically, by the body-mass trajectory, rather than by the behavioural test battery conventionally used in this paradigm; behavioural and biochemical endpoints should be integrated in the same animals in future work.

## 5 Conclusion

Candesartan pre-treatment reduced oxidative stress, enhanced antioxidant enzyme activities, restored TCA-cycle enzyme activities, and increased ATP concentration in both normal and UCMS-exposed rats. These findings provide a cellular and molecular basis for the beneficial biochemical effects of AT1R blockade on myocardial redox status and energy metabolism. Because blood pressure, cardiac function and systemic haemodynamic parameters were not measured, a contribution of blood-pressure-mediated mechanisms cannot be excluded, and the present data should be read as biochemical rather than functional evidence. They nonetheless support further evaluation of candesartan in the prevention of cardiovascular adverse effects of stress-related disorders such as anxiety and depression.

## Data Availability

The original contributions presented in the study are included in the article/Supplementary Material, further inquiries can be directed to the corresponding authors.
