# Supplementary material for: Effects of candesartan on cellular redox status and bioenergetics of cardiac tissue in normal and chronically stressed rats
Source: Front Pharmacol. 2026 Sep 15;17:1876050. doi: 10.3389/fphar.2026.1876050 (PMC13621241; doi:10.3389/fphar.2026.1876050)
Supplement: Supplementary file 1 [file Table1.docx]

**Table S1.** Oligonucleotide primers and quantitative real-time PCR assay characteristics for the antioxidant enzymes, TCA-cycle enzymes, and respiratory-chain complex subunits analyzed in rat myocardial tissue.

| **Gene symbol** | **Full name** | **RefSeq accession (with version)** | **Forward primer (5′→3′)** | **Reverse primer (5′→3′)** | **Amplicon size (bp)** | **Exon span** | **Primer Tm F / R (°C)** |
| --- | --- | --- | --- | --- | --- | --- | --- |
| *GPX1* | Glutathione peroxidase 1 | NM_030826 | AGTTCGGACATCAGGAGAATGG | AGGAAGGTAAAGAGCGGGTG | 148 | 339/340 (forward primer) | 59.83/ 59.39 |
| *SOD2* | Superoxide dismutase 2, mitochondrial | NM_017051 | GAACGGCCGTGTTCTGAGG | TTCTGCCCGCGCTGC | 80 | 106/107 (reverse primer) | 61.03/ 60.48 |
| *CAT* | Catalase | NM_012520 | TGCTCCCAACTACTACCCCA | AGAATGTCCGCACCTGAGTG | 140 | 1458/1459 (reverse primer) | 59.88/ 60.04 |
| *GR* | Glutathione-disulfide reductase | NM_053906 | GAACCAGATCCCAGGTGCC | AATGTAACCGGCACCCACAA | 100 | 360/361 (forward primer) | 60.08/ 60.18 |
| *GCL* | Glutamate–cysteine ligase, catalytic subunit | NM_012815.2 | CCGACCAATGGAGGTACAGTT | CGCTCCTGTGCCACTTTCAT | 150 | 1353/1354 (forward primer) | 59.72/60.96 |
| *GS* | Glutathione synthetase | NM_012962.1 | ATGCTGTCAGCCAGAACTCC | CAGGAACACAGTCTGGGCTA | 134 | 395/396 (reverse primer) | 60.04/59.02 |
| *CS* | Citrate synthase | NM_130755.1 | CCTTCCCGCCAGGTCCC | GCTCAGTATGTCTTTCAAATTCGTG | 145 | 164/165 (reverse primer) | 61.85/58.98 |
| *ICDH3* | Isocitrate dehydrogenase 3 (NAD⁺) alpha | NM_053638.2 | TGAGGGAAGTGGACGCGAT | TACTGTCTGAACGCCACCAG | 119 | 161/162 (reverse primer) | 60.98/59.68 |
| *SDH* | Succinate dehydrogenase complex flavoprotein subunit A | NM_130428.1 | GTTTCGAGACTTCTTCGCGG | ACTGGGTAGAAATCGCGTCTG | 121 | 167/168 (reverse primer) | 59.29/60.14 |
| *MDH* | Malate dehydrogenase 2, mitochondrial | NM_031151 | TGTGGCAGAGCTAAAGGGTT | GCTGGTCTTGGGGAAAGTCA | 128 | 657/658 (forward primer) | 59.23/59.89 |
| *NDUFS1* | NADH:ubiquinone oxidoreductase core subunit S1 (Complex I–III) | NM_001005550.1 | [AATCAGCGGGACAACTTGGT] | [TGCCAGTTGATCGAACATATCCT] | 117 | 123/124 (reverse primer) | 59.89/59.87 |
| *SDH* | Succinate dehydrogenase complex iron–sulfur subunit B (Complex II–III) | NM_001100539.1 | [TCTGCAATCCATCGAGGACC] | [CCAGCGATAAGCCTGCATGA] | 145 | 668/669 (reverse primer) | 59.53/60.53 |
| *ACTB* | Actin, beta (reference gene) | NM_031144.3 | GTCGAGTCCGCGTCCAC | TCATCCATGGCGAACTGGTG | 86 | 72/73 (reverse primer) | 60.18/60.39 |
